# Co-Fermentation of Edible Mushroom By-Products with Soybeans Enhances Nutritional Values, Isoflavone Aglycones, and Antioxidant Capacity of Douchi Koji

**DOI:** 10.3390/foods11192943

**Published:** 2022-09-20

**Authors:** Xiaoqin He, Peixiu Rong, Hongyan Liu, Bingcheng Gan, Dingtao Wu, Huabin Li, Renyou Gan

**Affiliations:** 1 Institute of Urban Agriculture, Chinese Academy of Agricultural Sciences, Chengdu National Agricultural Science & Technology Center, Chengdu 610213, China; 2 Key Laboratory of Coarse Cereal Processing (Ministry of Agriculture and Rural Affairs), Sichuan Engineering & Technology Research Center of Coarse Cereal Industralization, School of Food and Biological Engineering, Chengdu University, Chengdu 610106, China; 3 Guangdong Provincial Key Laboratory of Food, Nutrition and Health, Department of Nutrition, School of Public Health, Sun Yat-sen University, Guangzhou 510080, China

**Keywords:** Douchi koji, edible mushroom, protease, β-glucosidase, isoflavone, antioxidant activity

## Abstract

Douchi is a traditional salt-fermented soybean food with various bioactivities, such as anti-oxidation, anti-diabetes, and anti-hypertension, which are greatly affected by the activities of protease and β-glucosidase during koji production. Edible mushroom by-products are ideal ingredients for enhancing food flavor and nutritional quality due to their unique nutritional characteristics of high protein, rich amino acids, and low calories. However, there is no research on the preparation of Douchi by the mixed fermentation of edible mushroom by-products and soybeans. In this study, response surface methodology (RSM) was used to optimize the fermentation conditions of edible mushroom by-product Douchi koji (EMDK) with protease and β-glucosidase activities as indicators, and the changes in the main bioactive compounds and antioxidant activities of unfermented raw samples (URS), Douchi koji without edible mushroom by-product (DKWE), and EMDK were compared. The results of single-factor tests and RSM showed that the optimal fermentation conditions of EMDK were the *Aspergillus oryzae* to *Mucor racemosus* ratio of 1:1, inoculation amount of 6%, edible mushroom amount of 21%, and fermentation time of 63 h, and the activities of protease and β-glucosidase under these conditions were 796.03 ± 15.01 U/g and 1175.40 ± 36.98 U/g, respectively. Additionally, compared with URS and DKWE, the contents of total isoflavones and β-glucoside isoflavones in EMDK were notably decreased, while the contents of amino nitrogen, total phenolics, total flavonoids, and aglycone isoflavone, as well as the antioxidant capacity were significantly increased. Furthermore, significant correlations were found between the above components and antioxidant capacity. These results showed that edible mushroom by-product could be incorporated into soybeans for co-fermentation, conferring higher nutritional value to and antioxidant capacity of Douchi koji.

## 1. Introduction

Douchi, a traditional salt-fermented soybean food, has been widely consumed as a food condiment in China due to its attractive aroma, high nutritive value, and certain physiological activities, such as anti-oxidation, anti-diabetes, and anti-hypertension [1]. Generally, according to the predominant microorganisms involved in the koji-making stage, Douchi can be divided into four types, including *Mucor*-type, *Aspergillus*-type, *Bacterial*-type, and *Rhizopus*-type. Among them, *Aspergillus*-type and *Mucor*-type Douchi are the most popular Douchi in the market and have been produced in China for at least 2000 years [1,2]. It is worth noting that koji-making is a key step in producing high-quality and functional Douchi products. In this process, the enzyme system secreted by microbes plays a key role in the accumulation of nutrients and the transformation of components [3]. For example, most of the proteins in soybeans convert to peptone, polypeptide, and free amino acid through the enzymatic reactions catalyzed by proteinase, which is more conducive to human absorption [4]. In addition, β-glucosidase can convert isoflavonoid glycosides, one of the most important compounds with multitudinous health effects in soybeans, into aglycon isoflavonoids with a higher functional activity, thus endowing Douchi with better nutrition and health functions than soybeans [1,4]. Therefore, improving the activities of protease and β-glucosidase is the key to Douchi koji making.

Edible mushrooms are popular worldwide due to their health-beneficial properties (such as antioxidant activity), and unique nutritional characteristics of high protein, rich amino acids, and low calories [5,6]. However, it was estimated that nearly one-fifth of the by-product was produced during the mushroom production process, including caps, stalks, and deformed mushrooms, which not only leads to the waste of raw materials, but also causes certain environmental problems [7]. Therefore, various methods are being explored to increase the value-added product from mushroom waste to promote the circular economy and environmental protection [8]. Rathore et al. [9] reported that the supplementation of mushroom powder substantially enhanced the nutritional quality of cookies, including increased protein, fiber, minerals, phenolics, flavonoids, and antioxidants, and decreased starch hydrolysis and the glycemic index. Pasta with *Agaricus bisporus* powder showed an increase in moisture content, carbohydrates, total phenolic content, and antioxidant capacities [10,11]. These results support the mushroom powder as an ideal candidate for use in foods in efforts to improve their nutritional profiles. In addition, fermentation and enzymatic hydrolysis are considered to be effective methods to improve the sensory characteristics and nutritional components of food. The total phenolic content and antioxidant properties of *Pleurotus eryngii* fermented with selenium-enriched *Lactobacillus plantarum* were significantly improved [12]. In addition, it was reported that the antioxidant activity of *Pleurotus ostreatus* was significantly increased under the hydrolysis of proteinase K [13]. The by-products of edible mushrooms are rich in proteins, amino acids, polypeptides, flavonoids, and other nutritional active ingredients, which is very similar to the nutrients of soybeans [14]. Moreover, the abundant protease in the process of Douchi koji has the best enzymolysis effect on the flavor protein of edible mushrooms, which plays an important role in the preparation of more flavor peptides to enhance the flavor of food [15]. Therefore, we speculate that edible mushrooms will be a good partner to improve the nutritional quality and flavor of Douchi koji. Although Douchi has been produced in China for thousands of years, as far as we know, there are no reports of Douchi manufacturing by fermented edible mushroom byproducts coupled with soybeans. Therefore, the aim of this study was to focus on the optimal fermentation progress of the edible mushroom by-product Douchi koji (EMDK) with protease and β-glucosidase enzyme activities as indicators, and to compare the changes in the main bioactive compounds and antioxidant activities before and after fermentation.

## 2. Materials and Methods

### 2.1. Materials and Reagents

The soybeans, harvested in 2021, were purchased from Heilongjiang Changhe agricultural products processing Co., Ltd. (Hailun, China). The edible mushroom byproduct powder (EMBP, 60 mesh), including *White Hypsizygus marmoreu* and *Hypsizygus marmoreus*, were obtained from Chengdu Huiguyuan Biotechnology Co., Ltd. (Chengdu, China). The strains were obtained from the China Center of Industrial Culture Collection (CICC), including *Mucor racemosus* (CICC 40481), *Actinomucor elegans* (CICC 40252), *Mucor Wutungkiao* (CICC 3109), and *Aspergillus oryzae* (CICC 41736).

The chemicals, including daidzin (98%), genistin (98%), glycitin (98%), daidzein (98%), genistein (98%), glycitein (98%), Folin–Ciocalteu’s phenol reagent, 6-hydroxy-2,5,7,8-tetramethylchromane-2-carboxylic acid (Trolox), 2,2-azinobis (3-ethylbenothiazoline-6-sulfonic acid) (ABTS), and 2,2-diphenyl-1-picrylhydrazyl (DPPH) were purchased from Sigma-Aldrich (St. Louis, MO, USA). P-nitrophenyl-β-d-glucoside (p-NPG) and tyrosine were purchased from Tokyo Chemical Industry Co., Ltd. (Tokyo, Japan). All other chemicals were of reagent grade.

### 2.2. Selection of Fermented Strains and Preparation of Douchi Koji

The strains were first cultivated on bran seed medium at 28 °C for 3 days. Then, the spores produced were scraped into sterile water to obtain the spore suspension (1 × 10^7^ spores/mL). The preparation of Douchi koji was carried out with EMBP and soybeans as raw materials. The production process was slightly modified with reference to a previously reported method [16]. Briefly, cleaned soybeans were soaked in distilled water (1:3, m/v) at 35 °C for 4 h and mixed with 15% (m/m) EMBP evenly, and then steamed in an autoclave at 121 °C for 30 min (BKQ-B50II; Shandong Boke Biological Industry Co., Ltd., Jinan, China). Then, the cooling mixtures of soybeans and EMBP were inoculated with 4% (*v*/*w*) *Mucor racemosus*, *Actinomucor elegans*, *Mucor Wutungkiao*, and *Aspergillus oryzae* spore suspension (1 × 10^7^ spores/mL) as a pure culture, respectively, and fermented at 28 °C for 72 h in an incubator with a relative humidity of 80%.

### 2.3. Optimization of EMDK

#### 2.3.1. Single-Factor Tests

The effects of four independent factors on the activities of neutral protease and β-glucosidase of EMDK were tested through the principle of single variable, including the inoculation amount (2, 4, 6, 8, 10, 12, and 14%, v/m), strain proportion (the ratio of *Aspergillus oryzae* to *Mucor racemosus*, 6:1, 4:1, 2:1, 1:1, 1:2, 1:4, and 1:6, *v*/*v*), EMBP amount (0, 5, 10, 15, 20, 25, 30, 35%, m/m), and fermentation time (24, 36, 48, 54, 68, 72, and 96 h).

#### 2.3.2. Response Surface Methodology (RSM)

The RSM based on a three-level-three-variables Box–Behnken Design (BBD) was conducted to optimize the fermentation condition of EMDK. Three major variables that significantly affected the activities of neutral protease and β-glucosidase, including inoculation amount, EMBP amount, and fermentation time, were used as independent variables, and the coding of each variable was shown in Table 1.

### 2.4. Determination of Microbial Enzymes

#### 2.4.1. Neutral Protease Activity

The neutral protease activity of EMDK was measured based on the method reported by Deng et al. [3] with some modifications. Briefly, 1 g of EMDK was mixed with 10 mL phosphate buffer (pH = 7.5) and shaken at 120 rpm in an air bath at 37 °C for 30 min. The supernatant was collected as the enzyme assay sample after centrifugation for 10 min at 4000× *g*. Then, 1 mL of the preheated enzyme solution (40 °C, 2 min) was thoroughly mixed with 1.00 mL of 2% (m/v) casein, and incubated for 10 min at 40 °C. Next, 2 mL of 0.4 mol/L trichloroacetic acid was added to terminate the reaction. After standing for 10 min, 1 mL of supernatant was mixed with 5 mL of 0.4 mol/L sodium carbonate and 1 mL of Folin–Ciocalteu’s phenol reagent, and then incubated for 20 min at 40 °C. Finally, the absorbance of the mixture was measured at 680 nm.

#### 2.4.2. β-Glucosidase Activity

The β-glucosidase activity of EMDK was determined according to a previously reported method [17] with some modifications. Then, 2 g of samples were immersed in 10 mL of 0.2 mol/L acetate buffer (pH = 4.5) and extracted in an air bath shaker at 37 °C for 30 min. The supernatant was collected after centrifugation at 4000× *g* for 20 min. Then, 0.5 mL of crude enzyme solution and 2 mL of 1 mM/L p-NPG solution were mixed and incubated at 45 °C for 5 min. After that, 2.5 mL of 1 mol/L sodium carbonate was added immediately to stop the above reaction. Finally, the absorbance was detected at 400 nm.

### 2.5. Determination of Amino Nitrogen (AN) Contents

The contents of AN in the EMDK at the optimal fermentation conditions, the DKWE at the same conditions, and the URS were determined by Formaldehyde titration according to the method of Ko et al. [18].

### 2.6. Determination of Total Phenolics Content (TPC) and Total Flavonoid Content (TFC)

The TPC and TFC of the EMDK, DKWE, and URS were determined as described previously with slight modification [19]. Briefly, 1g freeze-dried sample was immersed in 15 mL of 60% (*v*/*v*) ethanol aqueous solution and extracted at 25 °C for 2 h. Then, the supernatant was collected after centrifugation at 4000× *g* for 20 min. The TPC was evaluated by the Folin-Ciocalteu method and expressed as mg gallic acid equivalent (GAE)/g dry weight (DW). The TFC was determined using the aluminum chloride colorimetric method and expressed as mg catechin equivalent (CE)/g DW.

### 2.7. Determination of Isoflavones Contents

The contents of isoflavones in the EMDK, DKWE, and URS were determined as described previously [16] with slight modifications. Briefly, 1 g freeze-dried sample was mixed with 10 mL of 80% (*v*/*v*) methanol and ultrasound treated at 37 °C for 60 min. Then, the centrifuged supernatant (4000× *g*, 15 min) was filtered through a 0.45 μm filter unit. High-performance liquid chromatography (HPLC) analysis equipped with an Agilent Zorbax SB-C18 column (4.6 × 150 mm, 5 µm) was employed to identify and quantify the isoflavones. The mobile phase for HPLC was composed of water containing 0.1% acetic acid (solvent A) and 0.1% (*v*/*v*) acetic acid in acetonitrile (solvent B). In addition, the elution program was carried out as follows: 15~30% solvent B for 0~20 min, 30~50% solvent B for 20~40 min, and 50~65% solvent B for 40~55 min. The column temperature, injection volume, flow rate, and detection wavelength were 40 °C, 10 µL, 0.7 mL/min, and 280 nm, respectively. Qualitative and quantitative data on each isoflavone were obtained by comparing their retention time and peak area of the known standard. Data were expressed in mg/g dry weight (DW).

### 2.8. Determination of Antioxidant Capacity

The DPPH radical scavenging, ferric-reducing antioxidant power (FRAP), and ABTS radical scavenging assays were adopted to evaluate the antioxidant activities of EMDK, DKWE, and URS according to our previous study [19].

### 2.9. Statistical Analysis

All experiments were conducted in parallel at least three times, and the results were presented as mean ± standard deviation (SD). One-way analysis of variance (ANOVA) plus post hoc Duncan’s multiple test were used to analyze the data by SPSS 20.0 software (SPSS Inc., Chicago, IL, USA), and statistical significance was defined at *p* < 0.05. Finally, Pearson’s correlation analysis was conducted by the programming language R (V4.0.0, AT&T Bell Laboratories, New Zealand).

## 3. Results and Discussion

### 3.1. Screening of Strains with High Neutral Protease and β-Glucosidase Activities

**Figure 1:**
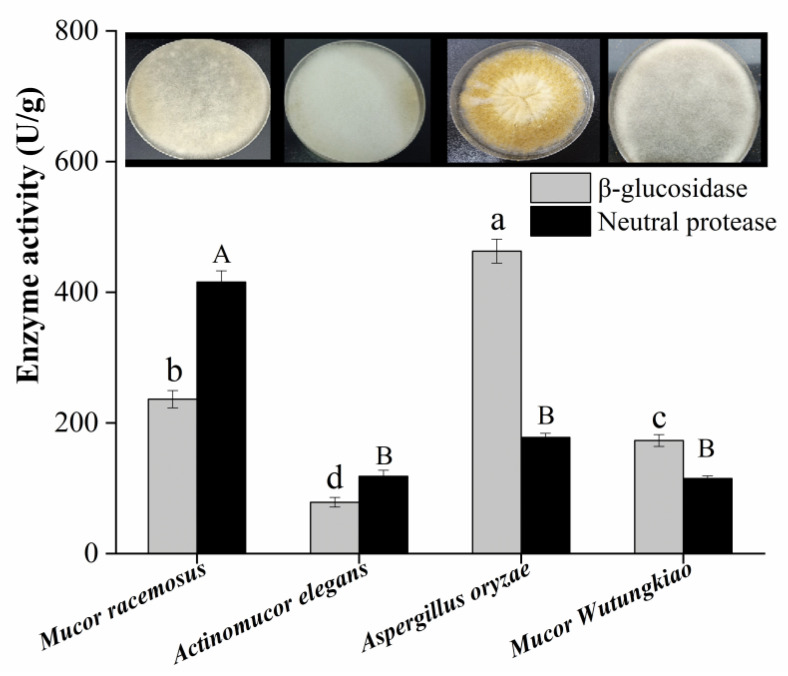
The activities of neutral protease and β-glucosidase of different strains. Values are expressed as the mean ± SD (*n* = 3). Bars with different uppercase or lowercase letters represent significant differences (*p* < 0.05).

The koji-making stage of Douchi is an important process of the accumulation of protease and β-glucosidase, which directly affects the quality and function of Douchi [3]. Therefore, four strains of fungi commonly used in Aspergillus-type and Mucor-type Douchi, including *Mucor racemosus* (CICC 40481), *Actinomucor elegans* (CICC 40252), *Mucor Wutungkiao* (CICC 3109), and *Aspergillus oryzae* (CICC 41736), were selected to evaluate the activities of protease and β-glucosidase. As shown in Figure 1, under the fermentation condition of 4% (v/m) inoculation amount, 15% (m/m) EMBP, and 72 h fermentation time, *Mucor racemosus* had the highest protease activity (436.32 ± 17.74 U/g), and *Aspergillus oryzae* showed the highest β-glucosidase activity (473.13 ± 31.65 U/g) (*p* < 0.05). He et al. [1] found that the amino acid content of *Mucor*-type Douchi was significantly higher than that of *Aspergillus*-type Douchi, which might be attributed to the more adequate proteolysis caused by the higher protease activity. Additionally, Liu et al. [20] found that the *Aspergillus*-type Douchi had more isoflavone aglycones content than that of *Mucor*-type and *Bacillus*-type Douchi, which might be related to the higher activity of β-glucosidase in *Aspergillus*-type Douchi. Therefore, combined with their respective advantages, *Mucor racemosus* and *Aspergillus oryzae* were selected as the subsequent fermentation strains.

### 3.2. Analysis of Single-Factor Tests

**Figure 2:**
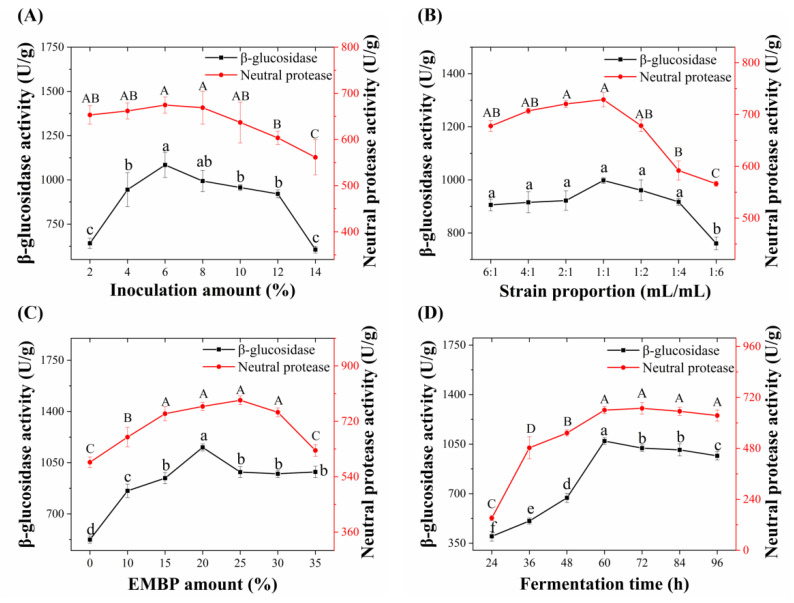
Effects of different inoculation amount (**A**); strain proportion (**B**); EMBP amount (**C**) and fermentation time (**D**) on the activities of neutral protease and β-glucosidase in EMDK. Values are expressed as the mean ± the SD (*n* = 3). Bars with different uppercase or lowercase letters represent significant differences (*p* < 0.05).

The single-factor experimental results (inoculation amount, strain proportion, EMBP amount, and fermentation time) of EMDK fermentation are shown in Figure 2. Briefly, under the fixed strain proportion (1:1, *v*/*v*), EMBP amount (15%, m/m), and fermentation time (72 h), the activities of β-glucosidase and protease of EMDK increased with the inoculation amount until 6% (v/m), after which they decreased significantly (*p* < 0.05) (Figure 2A). Additionally, under the fermentation condition of 6% (v/m) inoculation amount, 15% (m/m) EMBP amount, and 72 h fermentation time, the activities of β-glucosidase and protease of EMDK reached the maximum when the strain proportion was 1:1 (*v*/*v*), and then began to decrease significantly (*p* < 0.05) (Figure 2B). Furthermore, when the inoculation amount (6%, v/m), strain proportion (1:1, *v*/*v*), and fermentation time (72 h) were fixed, the β-glucosidase activity of EMDK with 20% (m/m) EMBP was significantly higher than that of other conditions, reaching a value of 1054.83 ± 14.09 U/g, and then decreased significantly (Figure 2C). In addition, the protease activity of EMDK reached the maximum value of 788.43 ± 24.14 U/g when the amount of EMBP was 25% (m/m), but there was no significant difference with 20% (m/m) (Figure 2C). Finally, when the inoculation amount was 6% (v/m), the strain proportion was 1:1 (*v*/*v*), and the amount of EMBP was 20% (m/m), the activities of β-glucosidase and protease increased with the extension of fermentation time until 60 h (Figure 2D). However, when the fermentation time was higher than 60 h, the enzyme activities decreased within a small range and remained basically constant (Figure 2D). Taken together, the above results demonstrated that different fermentation parameters had a significant impact on the enzyme activities of EMDK, and the optimal inoculation amount, strain proportion, EMBP amount, and, fermentation time were 6% (v/m), 1:1 (*v*/*v*), 25% (m/m), and 60 h, respectively.

### 3.3. Response Surface Methodology (RSM) Experiments and Verification

#### 3.3.1. Results of Box–Behnken Design (BBD)

On the basis of the above single-factor test results, three major factors significantly affected the neutral protease activity and β-glucosidase activity of EMDK, and their corresponding condition ranges were confirmed (Table 1). The results of the BBD matrix are shown in Table 2. Then, two quadratic polynomial equations describing the relationship between these three variables and enzyme activities were obtained through multiple regression fitting analysis of the data in Table 2:

Y_1_ = 791.65 − 7.47A + 12.28B + 26.43C − 1.79AB − 6.39AC − 9.90BC − 39.37A^2^ − 12.62B^2^ − 78.73C^2^

Y_2_ = 1160.73 + 7.63A + 6.59B + 121.51C + 28.05AB − 0.8662AC − 16.31BC − 56.46A^2^ − 82.57B^2^ − 192.71C^2^

where Y_1_ and Y_2_ refer to the neutral protease activity and β-glucosidase activity of EMDK, respectively; and X_1_, X_2_, and X_3_ refer to inoculation amount, EMBP amount, and fermentation time, respectively.

The ANOVA analysis of the developed model equations in Table 3 showed that the two models were significant (*p* < 0.05), while the ‘Lack of Fit’ terms were not significant (*p* > 0.05), which suggested that the regression equation fitted the experiment well. In addition, the coefficient of determination (R^2^) of neutral protease activity and β-glucosidase activity-fitting models were 0.9973 and 0.9983, respectively, indicating the goodness of fit of the regression models. The adjusted R^2^ (R^2^_Adj_) of neutral protease activity and β-glucosidase activity models were 0.9938 and 0.9961, respectively, and the coefficients of variation (C.V.%) were 0.5647 and 0.9029, respectively, indicating that the predicted values were highly precise with a good degree of reliability, and the fermentation conditions of EMDK could be predicted by them.

#### 3.3.2. Effects of Independent Variables on the Activities of Neutral Protease and β-Glucosidase

**Figure 3:**
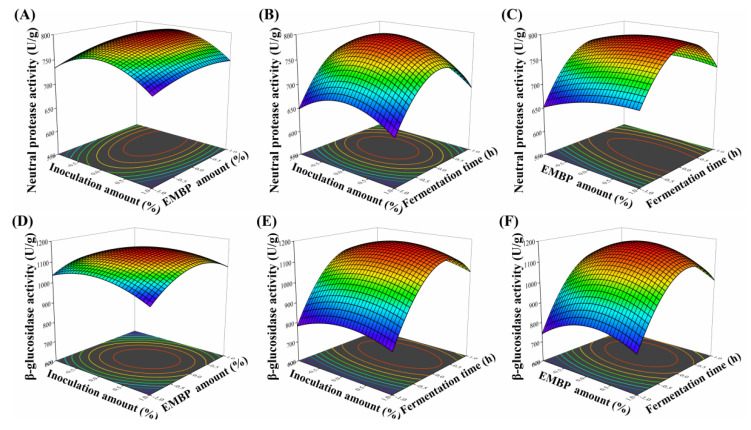
Three-dimensional response surface plots of independent variables on the activities of neutral protease (**A**–**C**) and β-glucosidase (**D**–**F**).

The three-dimensional response surface plots of independent variables on the activities of neutral protease and β-glucosidase are shown in Figure 3. The fermentation time (*p* < 0.0001), edible fungus powder amount (*p* < 0.0001), and the amount of inoculation (*p* = 0.0014) had a significant impact on the activity of neutral protease, and the interactions between the inoculation amount and fermentation time, as well as the EMBP amount and fermentation were obvious (*p* < 0.05). In addition, the inoculation amount and fermentation time also had a significant effect on the activity of β-glucosidase (*p* < 0.0001), while the EMBP amount was inconspicuous (*p* > 0.05). Moreover, it was obvious that the interactions between the inoculation amount and EMBP amount, as well as the EMBP amount and fermentation time, were significant (*p* < 0.05), while the interplay of the inoculation amount and fermentation time was not significant.

#### 3.3.3. Verification of the Model

The optimal fermentation conditions of EMDK obtained by the RSM experiment were an inoculation amount of 5.92%, an EMBP amount of 20.85%, and a fermentation time of 62.94 h, and the predicted values of protease activity and β-glucosidase activity were 795.00 U/g and 1176.82 U/g, respectively. According to the feasibility of the practical application, the conditions of the verification experiment were as follows: an inoculation amount of 6%; an EMBP amount of 21%; a fermentation time of 63 h, and the actual values of protease activity and β-glucosidase activity under those conditions were 796.03 ± 15.01 U/g and 1175.40 ± 36.98 U/g, respectively, which were close to the predicted values, indicating that the model was reliable and adequate. Furthermore, the protease activity and β-glucosidase activity of the EMDK under the optimal fermentation conditions were significantly increased by 34.92% and 121.43%, respectively, than those of DKEM under the same conditions, indicating that the addition of EMBP might increase the enzyme activities and promote the growth of *Mucor racemosus* and *Aspergillus oryzae*.

### 3.4. Analysis of Amino Nitrogen (AN) Contents

**Figure 4:**
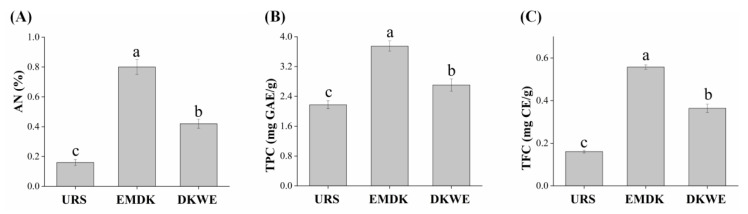
The amino nitrogen (AN), total phenolic content (TPC), and total flavonoid content (TFC) of different samples. (**A**) The amino nitrogen (AN) content; (**B**) The total phenolic content (TPC); (**C**) The total flavonoid content (TFC). Abbreviations: URS, unfermented raw samples; EMDK, edible mushroom by-product Douchi koji; DKWE, Douchi koji without edible mushroom by-product. Values are expressed as the mean ± SD (*n* = 3). Bars with different lowercase letters represent significant differences (*p* < 0.05).

The AN content, which can present the amino acid level, is one of the important physicochemical parameters to evaluate the fermentation degree and umami taste of Douchi [2]. Thus, the content of AN in URS, as well as the contents of AN in EMDK and DKWE at the optimal fermentation conditions were determined. As shown in Figure 4A, the content of AN in EMDK reached 0.82%, which was 3.90 times higher than that of URS. The above results were in agreement with Zhang et al. [2], who found that the AN content increased noticeably during the fermentation of natural-type and artificial-type Yongchuan Douchi. It was reported that the increase in the AN content might be related to the nitrogen compounds generated by the protein degradation in the raw materials [21]. Additionally, urea, a nonprotein nitrogenous compound, also plays a key role in increasing the AN content, through the decomposition of urease-producing microorganisms, such as *Pseudomonas* sp., into volatile compounds of ammonia [2,22]. Moreover, compared with DKWE, the content of AN in the EMDK was significantly increased by 0.89 times, indicating that the addition of edible mushroom might help improve the umami taste of Douchi koji. Umami substances are naturally found in a variety of foods, especially in mushrooms. It was reported that the umami flavor of mushrooms was mainly derived from peptides, amino acids, and other gustatory substances [23]. In this study, the wall-breaking effect of high-temperature and high-pressure cooking [24] and the enzymatic hydrolysis effect of neutral protease on flavoring protein [15] might be the reasons for promoting the release of flavoring substances, such as polypeptides and amino acids from edible mushroom proteins, and increasing the content of AN.

### 3.5. Analysis of TPC and TFC of Douchi Koji

It has long been recognized that the polyphenols and flavonoids in Douchi are the main material basis for the antioxidant and other beneficial effects on cancer inhibition [25]. Therefore, the TPC and TFC of URS, EMDK, and DKWE were determined. As shown in Figure 4B,C, EMDK showed the highest values of TPC (3.75 ± 0.14 mg GAE/g) and TFC (0.56 ± 0.01 mg CE/g), followed by DKWE, where the TPC and TFC were 2.70 ± 0.17 mg GAE/g and 0.36 ± 0.02 mg CE/g, respectively, while the URS showed the lowest TPC (2.18 ± 0.11 mg GAE/g) and TFC (0.16 ± 0.01 mg CE/g) values. Xu et al. [25] systematically assessed the bioactive substances of commercially fermented soy products, and found that the TPC (ranging from 7.17 to 12.37 mg GAE/g) and TFC (ranging from 0.64 to 1.23 mg CAE/g) in Douchi increased significantly after fermentation as compared with the raw yellow soybeans, which was consistent with our results. It was worth noting that the contents of TPC and TFC in this study were lower than those of the above commercially fermented soy products, which might be due to the short fermentation time of the koji preparation, and the post-fermentation process will be required to further accumulate and transform the polyphenols and flavonoids in Douchi. The phenolic compounds in soybeans exist mainly in insoluble form and covalently bind to the structural components of cell walls, such as cellulose, hemicellulose, lignin, pectin, and protein [26]. It was reported that the hydrolysis effect of enzymes secreted by microorganisms during fermentation, such as cellulase and amylase, was considered a possible strategy for converting insoluble phenolic compounds into free forms, thereby increasing the content of TPC and TFC [27]. Additionally, the destruction of material cell walls by cooking and enzymatic hydrolysis could increase the dissolution and extraction of phenolic compounds, thus improving the release of TPC and TFC [27], which may be another potential cause of increased TPC and TFC. Moreover, the substantial amount of phenols and flavonoids, such as chlorogenic, gallic, caffeic, protocatechuic, and syringic acids presenting in the edible mushroom [6], might partly account for the increased TPC and TFC recorded in EMDK when compared with DKEW. It is noteworthy to mention that the fermentation techniques have been reported to increase the content of phenolic acids in mushrooms [12], which might play a role in the increase of TPC and TFC in EMDK, but further studies are needed to verify and reveal the specific changes.

### 3.6. Analysis of Isoflavones Contents of Douchi Koji

**Figure 5:**
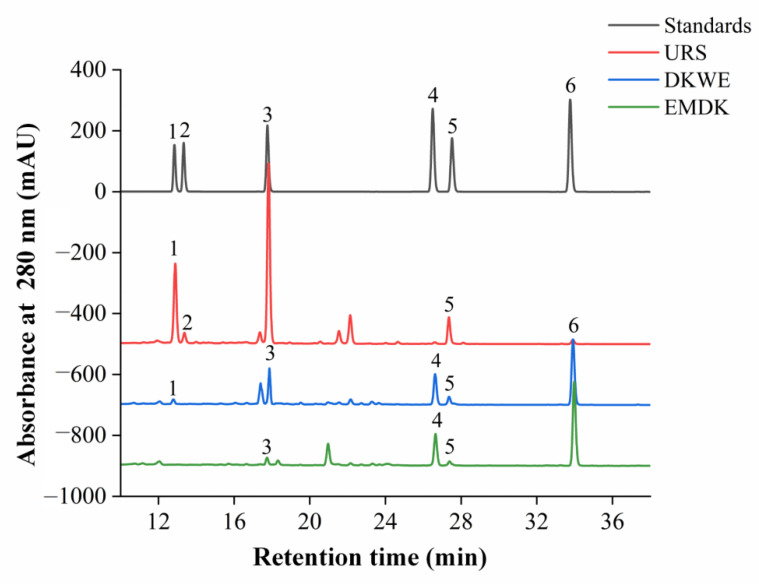
The HPLC chromatograms of standards and different samples at 280 nm. Abbreviations: URS, unfermented raw samples; EMDK, edible mushroom by-product Douchi koji; DKWE, Douchi koji without edible mushroom by-product. The labeled number represents the compound: 1, daidzin; 2, glycitin; 3, genistin; 4, daidzein; 5, glycitein; and 6, genistein.

Among all of the bioactive components, isoflavones are the most important compounds attributed to the healthful effects of inhibiting oxidative damage, lowering rates of breast cancer, and preventing osteoporosis in Douchi, and these effects mainly depend on the contents and structure of isoflavones [20]. It was reported that isoflavones in soybeans mainly existed in the forms of glucosides, including daidzin, glycitin, and genistin. However, in the process of soybean food fermentation, isoflavone glucosides could be hydrolyzed into their corresponding aglycones with higher biological activities under the key enzyme of β-glucosidase [16]. Therefore, both the content and composition of isoflavones, two important factors affecting the physiological function of Douchi, were determined in our study. As shown in Figure 5 and Table 4, compared with URS, the contents of total isoflavones and β-glucoside isoflavones in EMDK, including daidzin, glycitin, and genistin, were significantly reduced, while the contents of aglycones isoflavones, including daidzein, glycitein, and genistein were increased by 47.49, 4.41, and 10.15 times, respectively. Several studies reported that the content of isoflavone aglycones in soybean foods such as douchi, tofu, miso, natto, and tempeh increased significantly during the fermentation process [16,25], which was consistent with our results, indicating that the isoflavone glycosides were effectively transformed into their corresponding aglycones. Wang et al. [16] reported that the content and composition of isoflavones in soybean foods were not only related to the raw materials, but also mainly depended on the processing technologies, such as heat treatment, defoaming, enzyme hydrolysis, and fermentation. Thus, the soaking, cooking, and fermentation processes during koji preparation might be related to the change in isoflavone composition. In addition, it was suggested that the transformation of isoflavones related to microbial hydroxylation might be one of the reasons for the loss of isoflavones during the fermentation of Douchi [16]. Moreover, compared with DKWE, the addition of EMBP significantly increased the content of aglycones isoflavones in EMDK, speculating that edible mushroom might help to improve the antioxidant capacity of Douchi koji, but further studies are needed to explore the reasons for the increase in isoflavone aglycones.

### 3.7. Analysis of Antioxidant Capacity of Douchi Koji

**Figure 6:**
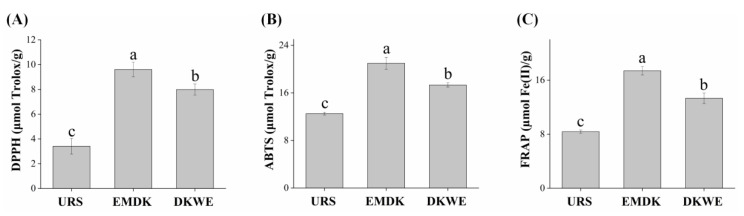
The antioxidant capacities of different samples. (**A**) The DPPH radical scavenging capacity; (**B**) The ABTS radical scavenging capacity; (**C**) The ferric-reducing antioxidant power (FRAP). Abbreviations: URS, unfermented raw samples; EMDK, edible mushroom by-product Douchi koji; DKWE, Douchi koji without edible mushroom by-product. Values are expressed as the mean ± the SD (*n* = 3). Bars with different lowercase letters represent significant differences (*p* < 0.05).

In recent years, increasing attention has been directed to the physiological properties of fermented soybean foods, such as the antioxidative, antiproliferative, and anti-hypertensive effects [16]. Among them, the antioxidant effect of Douchi has been well demonstrated [25]. In general, the antioxidant capacity of functional food depends not only on its active ingredients but also on the testing system [28]. Therefore, it is necessary to conduct multiple types of antioxidant capacity measurement instead of a single method for comprehensive evaluation. Currently, various methods, including free radical scavenging, lipid peroxidation inhibition, and metal ion chelation, have been widely investigated to evaluate the antioxidant potential [29]. Among them, DPPH and ABTS radicals are typical methods used to test the ability of free radical scavengers, hydrogen donors, or chain-breaking antioxidants [4], while FRAP assay is an ordinary and reproducible method for assessing the ability to reduce ferric (III) to ferrous (II) ions [28]. Thus, the antioxidant activities of URS, EMDK, and DKWE were assessed by the DPPH, ABTS, and FRAP assay in this study. As illustrated in Figure 6, the DPPH, ABTS, and FRAP values of EMDK were 1.81-, 0.68-, and 1.08-fold higher than those of URS, and 0.20, 0.21, and 0.31 times higher than DKWE, respectively, indicating that the fermentation process effectively endowed the raw material with a high antioxidant capacity [30,31]. Several studies had emphasized that the fermentation process was an effective method for increasing the antioxidant activity of food materials. As reported by Fan et al. [4], the ABTS radical and DPPH radical scavenging activities of Douchi increased from the beginning and reached a plateau at 16~20 h of fermentation. Xu et al. [25] also reported that a significant increase in the DPPH and FRAP values was found in the Douchi (23.05 μmol TE/g and 1.16 mmol FE/100 g, respectively) when compared with the raw yellow soybeans (1.52 μmol TE/g and 0.39 mmol FE/100 g, respectively). These reports were consistent with our results.

### 3.8. Correlation Analysis

**Figure 7:**
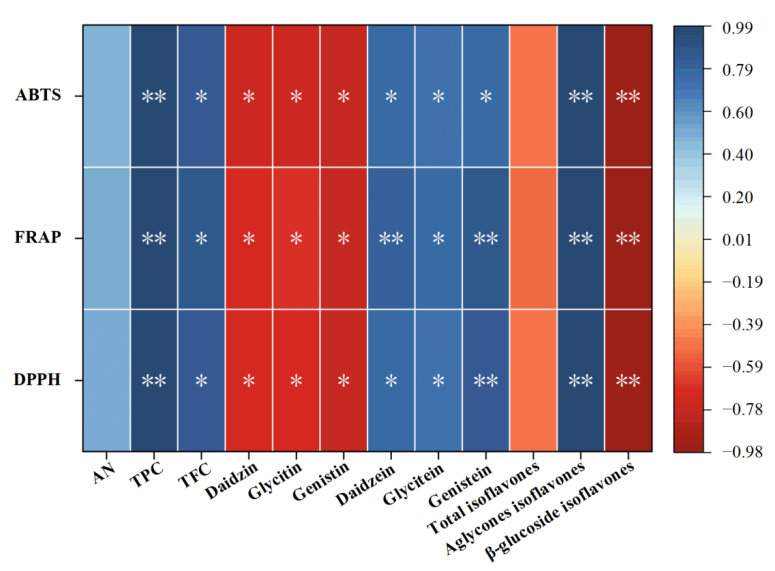
Correlation of the bioactive components and the antioxidant capacity of Douchi koji (* *p* < 0.05, and ** *p* < 0.01).

The improvement of antioxidant profiles in soy products after fermentation has sparked great attention [16]. Therefore, correlation analysis was conducted between the bioactive components and the antioxidant capacity of Douchi koji to further explore the possible internal relationship. As the heatmap showed in Figure 7, the FRAP, ABTS, and DPPH values of Douchi koji were positively correlated with the contents of TPC, TFC, and aglycones isoflavones (daidzin, glycitin, and genistin), but negatively correlated with the content of β-glucoside isoflavones (daidzin, glycitin, and genistin), suggesting that phenolic acid, flavonoids, and aglycones isoflavones might be the main material basis for the antioxidant effect of Douchi koji. Xu et al. [25] compared the physicochemical indexes of different commercially fermented soybean products and found that the antioxidant capacity of Douchi was positively correlated with the levels of TPC and TFC. In addition, the samples containing a high content of aglycone isoflavones possessed higher overall antioxidant capacity [25], which was consistent with our results. Overall, the increased contents of antioxidant substances, including TPC, TFC, and aglycone isoflavones after fermentation might be the main reason for the improvement of antioxidant activity of Douchi koji, which might help to protect the body from free radical attack and further reduce the risk of many chronic diseases, such as cancer and cardiovascular diseases [27].

## 4. Conclusions

In conclusion, this study found that the optimal fermentation conditions of EMDK were the *Aspergillus oryzae* to *Mucor racemosus* ratio of 1:1, inoculation amount of 6%, edible mushroom amount of 21%, and fermentation time of 63 h, and the activities of neutral protease and β-glucosidase, respectively, were 796.03 ± 15.01 U/g and 1175.40 ± 36.98 U/g under those conditions. In addition, compared with URS and DKWE, the antioxidant capacity of EMDK was notably increased, which was positively correlated with the increase in AN, TPC, TFC, and aglycone isoflavones, but negatively correlated with the decrease in β-glucoside isoflavones. Therefore, co-fermentation with edible mushroom by-products and soybeans may be a feasible strategy to improve both the nutritional value and antioxidant activity of Douchi koji.

## Figures and Tables

**Table 1:** Box–Behnken design.

Independent Variable	Units	Code Levels		
		−1	0	1
Inoculation amount (X_1_)	% (mL/g)	4	6	8
EMBP amount (X_2_)	% (g/g)	15	20	25
Fermentation time (X_3_)	h	48	60	72

**Table 2:** Experimental results for Box–Behnken design.

Runs	X_1_	X_2_	X_3_	Y_1_	Y_2_
	Inoculation Amount (%)	EMBP Amount (%)	Fermentation Time (h)	Neutral Protease Activity (U/g)	β-Glucosidase Activity (U/g)
1	−1	0	−1	648.942	787.45
2	−1	−1	0	733.564	1040.33
3	0	0	0	793.387	1166.42
4	0	1	1	730.342	1007.93
5	0	0	0	792.987	1155.38
6	1	0	−1	642.809	805.419
7	−1	0	1	717.076	1019.45
8	0	−1	1	720.627	1018.75
9	1	1	0	742.187	1059.18
10	−1	1	0	756.72	988.789
11	0	−1	−1	650.453	730.366
12	0	0	0	788.187	1164.94
13	0	1	−1	699.787	784.774
14	0	0	0	789.787	1156.1
15	1	−1	0	726.187	998.538
16	1	0	1	685.387	1033.95
17	0	0	0	793.92	1160.82

**Table 3:** The ANOVA analysis of the quadratic models.

Response Value	F-Value	*p*-Value	Significant
Neutral protease			
Model	284.07	<0.0001	significant
Lack of Fit	4.91	0.0792	not significant
R2	0.9973		
R2Adj	0.9938		
C.V.%	0.5647		
Model	284.07	<0.0001	significant
Lack of Fit	4.91	0.0792	not significant
β-glucosidase			
Model	455.88	<0.0001	significant
Lack of Fit	6.33	0.0533	not significant
R2	0.9983		
R2Adj	0.9961		
C.V.%	0.9029		
Model	455.88	<0.0001	significant
Lack of Fit	6.33	0.0533	not significant

**Table 4:** The contents of isoflavones (µg/g dry matter).

Samples	β-Glucoside			Aglycones			Total Isoflavones
	Daidzin	Glycitin	Genistin	Daidzein	Glycitein	Genistein	
URS	583.88 ± 5.75	69.85 ± 1.51	567.48 ± 7.95 ^a^	4.95 ± 0.21 ^c^	11.95 ± 0.40 ^c^	31.66 ± 0.52 ^b^	1269.77 ± 15.13 ^a^
EMDK	nd	nd	2.47 ± 0.68 ^b^	240.04 ± 4.12 ^a^	64.60 ± 1.60 ^a^	353.12 ± 4.16 ^a^	660.22 ± 8.17 ^b^
DKWE	3.12 ± 0.51	nd	16.24 ±0.33 ^b^	121.39 ± 1.70 ^b^	43.11 ± 0.85 ^b^	271.20 ± 3.72 ^a^	455.06 ± 5.62 ^b^

Abbreviations: URS, unfermented raw samples; EMDK, edible mushroom by-product Douchi koji; DKWE, Douchi koji without edible mushroom by-product. Data are expressed as the mean ± the SD (*n* = 3). The different superscript lowercase letters in the same column indicate statistical significance (*p* < 0.05).

## Data Availability

The data presented in this study are available on request from the corresponding author.

## Abbreviations

Edible mushroom by-product Douchi koji, EMDK; Unfermented raw samples, URS; Douchi koji without edible mushroom by-product, DKWE; edible mushroom byproduct powder, EMBP; Response surface methodology, RSM; Box–Behnken Design, BBD; Amino nitrogen, AN; Total phenolics content, TPC; Total flavonoid content, TFC; High-performance liquid chromatography, HPLC; Ferric-reducing antioxidant power assay, FRAP; 3-ethylbenothiazoline-6-sulfonic acid, ABTS; 2,2-diphenyl-1-picrylhydrazyl, DPPH.
